# High-Affinity Anti-VISTA Antibody Protects against Sepsis by Inhibition of T Lymphocyte Apoptosis and Suppression of the Inflammatory Response

**DOI:** 10.1155/2021/6650329

**Published:** 2021-07-28

**Authors:** Tianzhu Tao, Lulong Bo, Teng Li, Longbao Shi, Hui Zhang, Bo Ye, Yuhai Xu, Qingqing Ma, Xiaoming Deng, Guorong Zhang

**Affiliations:** ^1^Department of Anesthesiology, Air Force Medical Center, Beijing 100142, China; ^2^Department of Pain Medicine, Air Force Medical Center, Beijing 100142, China; ^3^Faculty of Anesthesiology, Changhai Hospital, Shanghai 200433, China; ^4^Department of Pathology, Air Force Medical Center, Beijing 100142, China; ^5^Department of Neurosurgery, Air Force Medical Center, Beijing 100142, China

## Abstract

**Background:**

B7 family members and ligands have been identified as critical checkpoints in orchestrating the immune response during sepsis. V-domain Ig suppressor of T cell activation (VISTA) is a new inhibitory immune checkpoint involved in restraining T cell response. Previous studies demonstrated that VISTA engagement on T cells and myeloid cells could transmit inhibitory signals, resulting in reduced activation and function. The current study was designed to determine the potential therapeutic effects of a high-affinity anti-VISTA antibody (clone MH5A) in a murine model of sepsis.

**Methods:**

Polymicrobial sepsis was induced in male C57BL/6 mice via cecal ligation and puncture. Expression profiles of VISTA on T lymphocytes and macrophage were examined at 24 and 72 h postsurgery. The effects of anti-VISTA mAb on the 7-day survival, lymphocyte apoptosis, cytokine expression, bacterial burden, and vital organ damage were determined. Furthermore, the effects of anti-VISTA mAb on CD3^+^ T cell apoptosis and macrophage activation were determined *in vitro*.

**Results:**

VISTA was substantially expressed on T cells and macrophages in sham-operated mice; septic peritonitis did not induce significant changes in the expression profiles. Treatment with MH5A improved the survival of septic mice, accompanied by reduced lymphocyte apoptosis, decreased cytokine expression, and enhanced bacterial clearance. Engagement of VISTA receptor with MH5A mitigated CD3^+^ T cell apoptosis cultured from CLP mice and suppressed LPS-induced cytokine production by macrophage *in vitro*.

**Conclusion:**

The present study identified VISTA as a novel immune checkpoint in the regulation of T cell and macrophage response during sepsis. Modulation of the VISTA pathway might offer a promising opportunity in the immunotherapy for sepsis.

## 1. Introduction

Sepsis is defined as a life-threatening organ dysfunction that is attributed to the dysregulated host response to infections [1]. In recent years, despite advances in the treatment and management, the morbidity and mortality of patients suffering from sepsis remain unacceptably high [2]. The increasing understanding of the nature of immune response during infections has resulted in a paradigm shift in our view of the pathogenesis. Sepsis was considered to be an overwhelming proinflammatory response that was followed by a phase of protracted long-term immunosuppression, which was characterized by profound depletion of immune cells, expansion of Tregs and myeloid-derived suppressor cells, and secondary infections [3, 4]. Inhibitory immune checkpoints are negative regulators of immune activation and have been implicated in sepsis-induced immune dysfunction [5]. Immune checkpoint inhibitors have recently been emerged as a novel therapy in improving the host immunity against cancer, which is highly encouraging to the immunotherapeutic strategies against sepsis [6]. Recently, animal studies demonstrated that blockade of the CTLA-4 or PD-1/PD-L1 pathway improved survival in several clinically relevant models of sepsis, which highlighted the significant degree of coinhibitory molecules in sepsis-induced immune dysfunction [7].

V-domain Ig suppressor of T cell activation (VISTA) is a new member of the B7 family that possesses high homology to PD-L1 and is exclusively expressed on the hematopoietic cells. VISTA has been identified as an immune checkpoint molecule that negatively regulates T cell activation. A soluble VISTA Ig protein or VISTA expression on antigen-presenting cells (APCs) acts as a ligand which suppresses T cell proliferation and cytokine production [8]. Neutralizing anti-VISTA antibody could reverse VISTA-mediated T cell dysfunction [9]. Disruption of VISTA lowers the threshold of T cell activation, allowing for an enhanced proinflammatory phenotype and an increase in the intensity of autoimmune diseases [10]. Further, Han et al. [11] reported that VISTA expression on neutrophils and pDCs could also transmit inhibitory signals, resulting in reduced cytokine expression under stimulation. Together, these studies suggested that VISTA expression exerts nonredundant regulatory functions on the activation of lymphocytes and myeloid subsets. The observation that VISTA functions to restrain T cell and myeloid cell activation led to our hypothesis that modulation of the VISTA pathway might preserve or aggravate the host immunity during sepsis. To test this hypothesis, we conducted the present study to determine the specific role of VISTA in sepsis-associated immune dysfunction and explore the potential underlying mechanisms.

## 2. Materials and Methods

### 2.1. Animals and CLP Models

Male C57BL/6 mice aged 8-12 weeks were obtained from the Animals Experimentation Center of Naval Medical University. Animals were kept in the laboratory of Changhai Hospital for at least one week prior to the initiation of the animal experiments. The study protocols were approved by the Institutional Animal Care and Use Committee of Changhai Hospital.

A CLP model of sepsis was induced as described before [12]. Briefly, mice were anesthetized with 2-3% isoflurane, and then, a 1 cm midline incision was made. The cecum was exposed and ligated distally, and punctured twice with a 22-gauge needle. Mice in the sham group had the cecum externalized but without ligation or puncture. The abdominal wall was closed in two layers, and then, each mouse was subcutaneously injected with 1 mL lactated Ringer's solution. Mice were allowed free access to food and water throughout the experiment.

### 2.2. Reagents and Treatment

The following antibodies were purchased from BioLegend (CA, USA): CD3-APC (clone 17A2), CD4-APC (clone RM4-4), CD8-FITC (clone 53-6.7), and VISTA-PE (clone MH5A). TNF-*α* and LPS were purchased from Sigma (St. Louis, MO, USA). Anti-cleaved caspase-3 (Asp175) antibody and anti-*β*-actin antibody for Western blotting assay were purchased from Cell Signaling Technology (Danvers, MA, USA). Purified anti-CD3 antibody and anti-CD28 antibody were obtained from eBioscience (San Diego, CA, USA). Purified anti-VISTA antibody (clone: MH5A, BioLegend) or isotype control (clone: HTK888, BioLegend) was intravenously administered at 100 *μ*g/100 *μ*L 1 h after CLP procedures.

### 2.3. Flow Cytometry for the Determination of VISTA Expression

Mice that underwent CLP or sham operation were sacrificed 24 or 72 h postsurgery. Samples of the blood and spleens were harvested, and then stained with anti-CD4, anti-CD8, and/or anti-VISTA antibodies. Flow cytometry was performed using MACSQuant Analyzer (Miltenyi Biotec, Bergisch Gladbach, Germany), and data were analyzed using Flowjo software version 10.0 (Tree Star, Ashland, OR, USA).

### 2.4. Determination of Apoptosis in the Thymus and Spleen

The thymus and spleen were harvested 24 h after CLP or sham surgery. Then, single-cell suspensions of the thymus were prepared and stained with anti-CD3, annexin V, and propidium iodide (PI). Annexin V-positive cells were gated and considered apoptotic events. Splenic apoptosis was determined by using terminal deoxynucleotidyl transferase-mediated dUTP nick end labeling (TUNEL) staining (ApopTag Plus Peroxidase In Situ Apoptosis Detection Kit, Chemicon, Billerica, MA, USA). Briefly, sections were incubated in the equilibration buffer for 10 min and then stained with terminal deoxynucleotidyl transferase and dUTP-digoxigenin. The reaction was stopped after incubation for 1 h at 37°C. The slices were washed followed by the addition with anti-digoxigenin-peroxidase solution. The sections were then colorized with DAB/H_2_O_2_ and counter-stained with bis-benzamide. For each specimen, five fields per section were randomly examined at a higher magnification (400x). Two investigators examined the samples independently, and the mean density of the TUNEL-positive cells was used to assess the apoptosis.

### 2.5. Cytokine Analysis

Blood samples were harvested from septic or sham-operated mice 24 hours after treatment. Plasma tumor necrosis factor (TNF-*α*), IL-6, IL-10, and interferon-*γ* (IFN-*γ*) were measured using the murine enzyme-linked immunosorbent assay (ELISA) kit (BD Biosciences, CA, USA) following the manufacturer's instructions.

### 2.6. Determination of the Bacterial Burden

Mice that underwent CLP were treated with MH5A antibody or isotype control and euthanized 24 h postsurgery. Blood samples were prepared with dilutions of 10^3^ or 10^5^ in 100 *μ*L saline and spread on tryptic soy agar with 5% sheep blood agar plate. Likewise, peritoneal lavage fluid was collected after washing the peritoneum in 2 mL sterile PBS and serially diluted to 10^5^ or 10^7^ fold. Plates were incubated at 37°C for 24 h, and colony counts were expressed as colony forming units per milliliter (CFU/mL).

### 2.7. Histopathologic Determination

Samples of the spleen, liver, and lung were collected 24 h postsurgery. Samples were fixed with 10% neutral buffered formaldehyde and embedded in paraffin. Sections were prepared and stained with the hematoxylin-eosin reagent, following which two experienced pathologists examined the sections separately.

### 2.8. Survival Study

Mice that underwent sham operation or CLP were randomly selected to receive a bolus intravenous injection of MH5A mAb (100 *μ*g), isotype control (100 *μ*g), or saline (100 *μ*L) 1 h postsurgery with a total of 12 mice in each group. Survival rate was assessed over seven subsequent days.

### 2.9. Anti-VISTA mAb on T Cell Apoptosis In Vitro

Spleen samples were harvested 24 h post-CLP or sham surgery. Single cell suspension of the spleen was prepared after lysis of erythrocytes. CD3^+^ T cells were enriched from CLP or sham-operated mice with a purity of over 90% by using a CD3^+^ T cell isolation kit (Miltenyi Biotec, Germany). Purified T cells were cultured at 1 × 10^6^/mL in 96-well plates with the presence of 10 *μ*g/mL anti-CD3 antibody and 5 *μ*g/mL anti-CD28 antibody. To determine the role of the VISTA pathway in the regulation of T cell apoptosis, MH5A (10 *μ*g/mL) or isotype control (10 *μ*g/mL) was added to the CD3^+^ T cells with or without the presence of TNF-*α* (1 *μ*g/mL). Cells were collected after 48 h incubation and then stained with annexin V and PI, and apoptotic cells were determined by flow cytometry. Further, cleaved caspase-3 was determined by Western blotting assay, and *β*-actin was used as the internal control.

### 2.10. Anti-VISTA mAb on Cytokine Expression by Macrophages

Peritoneal macrophages were elicited by intraperitoneally injection of sodium periodate 3 d before harvest. The cells were seeded at 5 × 10^6^/mL per well and cultured in RMPI-1640 (HyClone; Thermo Scientific, Waltham, MA) supplemented with 10% fetal bovine serum overnight. Macrophages were pretreated with MH5A mAb (10 *μ*g/mL), isotype control (10 *μ*g/mL), or PBS for 2 hours and then challenged with LPS (10 ng/mL) for 24 hours. Supernatant was collected and cytokine expressions of TNF-*α*, IL-6, and IL-10 were examined by ELISA as described.

### 2.11. Statistical Analysis

Data are expressed as the mean ± SEM and analyzed using Mann-Whitney or analysis of variance (ANOVA), as appropriate. Bonferroni test was used as a post hoc test for the correction of multiple comparisons. Log-rank test was performed to determine the significance of difference between the groups in the survival studies. Statistical analysis was conducted using Prism 6.0 (GraphPad Software, San Diego, CA). Statistical significance was accepted at *P* < 0.05.

## 3. Results

### 3.1. VISTA Expression on T Cells and Macrophage

VISTA expressions on T cells and macrophage were determined at 24 h or 72 h post-CLP or sham surgery. VISTA was substantially expressed on CD4^+^ T cells and CD8^+^ T cells in both the spleen and peripheral blood in the sham group. Septic peritonitis did not induce significant changes of VISTA expression on either CD4^+^ or CD8^+^ T cells. VISTA was also highly expressed on the macrophage from the spleen and peritoneum, which was maintained at a high level following septic insult (see Supplement Figure 1).

### 3.2. Anti-VISTA mAb Improved the 7-Day Survival of Septic Mice

**Figure 1:**
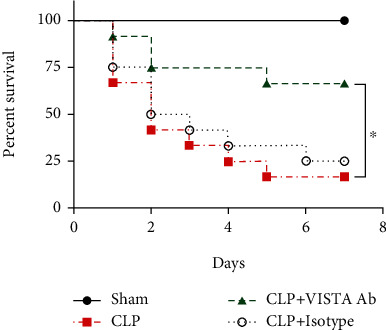
Anti-VISTA mAb improved a 7-day survival in septic mice. CLP mice were treated with MH5A antibody (100 *μ*g), isotype control (100 *μ*g), or saline 1 h postsurgery. Data are shown as percentage survival after 7 days. Survival study was performed with 4 mice in each group and repeated thrice (*n* = 12 in each group).^∗^*P* < 0.05.

To evaluate the potential therapeutic effects of the anti-VISTA mAb in septic mice, a 7-day survival study was conducted. All sham-operated mice survived during the 7 days, while the survival rate in the CLP group was 16.7% (2/12). Treatment with MH5A mAb showed a significantly higher survival rate (66.7%, 8/12) as compared to that in the control group (*P* = 0.01) (Figure 1).

### 3.3. Anti-VISTA mAb Alleviated the Vital Organ Injury following Sepsis

**Figure 2:**
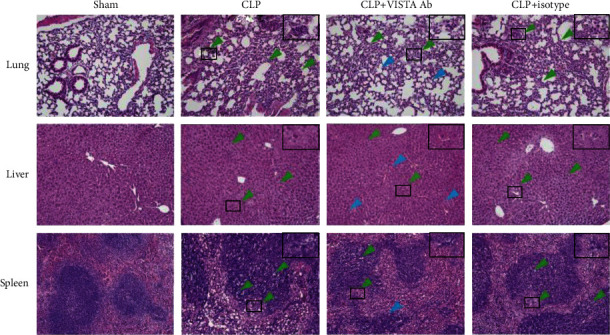
Histopathological lesions were determined in the lung, liver, and spleen following sepsis. Representative images showed improvement in the vital organ injury in mice treated with MH5A antibody. Sections were examined under the microscope at a magnification of 200x by two separate pathologists. The green arrows indicate histopathological lesions, and the blue arrows refer to normal pathological appearance.

Vital organ injury was determined in the lung, liver, and spleen at 24 h post-CLP or sham surgery. As shown in Figure 2, sepsis induced substantial pathological lesions in each organ. Pathological changes in the lung were observed as seen by thickening of the alveolar wall, infiltration of inflammatory cells, and impaired alveoli following CLP. Liver injury was evidenced by swollen hepatocytes and absent hepatic sinusoids. Vascular leakage and cellular apoptosis following polymicrobial infections in the spleen were observed. Anti-VISTA mAb largely alleviated the pathological lesions in vital organs as compared to that in the control group.

### 3.4. Anti-VISTA mAb Suppressed the Host Inflammatory Response and Enhanced Bacterial Clearance

**Figure 3:**
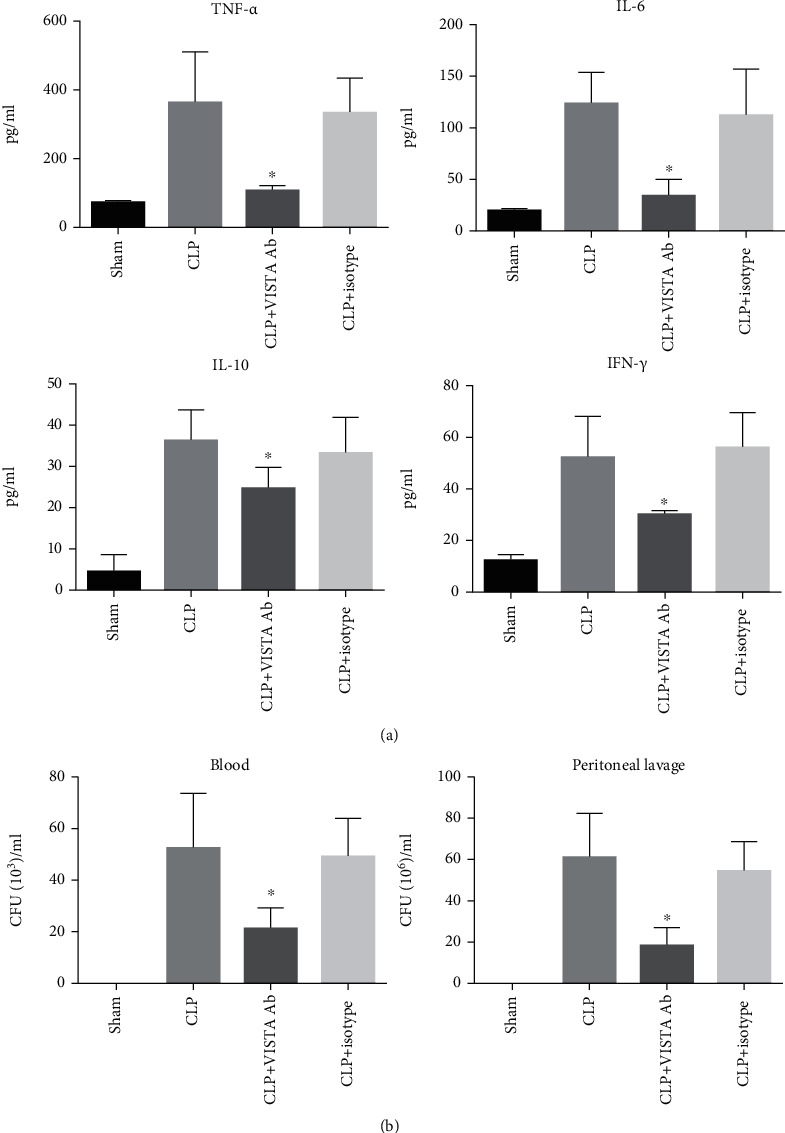
Anti-VISTA mAb inhibited the inflammatory response and enhanced bacterial clearance. (a) MH5A suppressed the expression of TNF-*α*, IL-6, IL-10, and IFN-*γ* at 24 h post-CLP. (b) MH5A reduced the bacterial burden in peripheral blood and at the infection site. All presented data are a composite of three independent experiments (*n* = 6 − 8 in each group). ^∗^*P* < 0.05.

Mice challenged with septic peritonitis had significantly higher plasma levels of TNF-*α*, IL-6, IL-10, and IFN-*γ* compared to the sham-operated mice. Treatment with anti-VISTA antibody markedly suppressed these cytokine expressions, as compared to that in the control group. Subsequently, bacterial burden was determined to evaluate the capacity of bacterial clearance. At 24 h postsurgery, lower levels of bacterial burden were seen both in the peripheral blood and at infectious site following the treatment with MH5A antibody (Figure 3(b)).

### 3.5. Anti-VISTA mAb Prevented the Apoptosis of Lymphocytes in both the Thymus and Spleen

**Figure 4:**
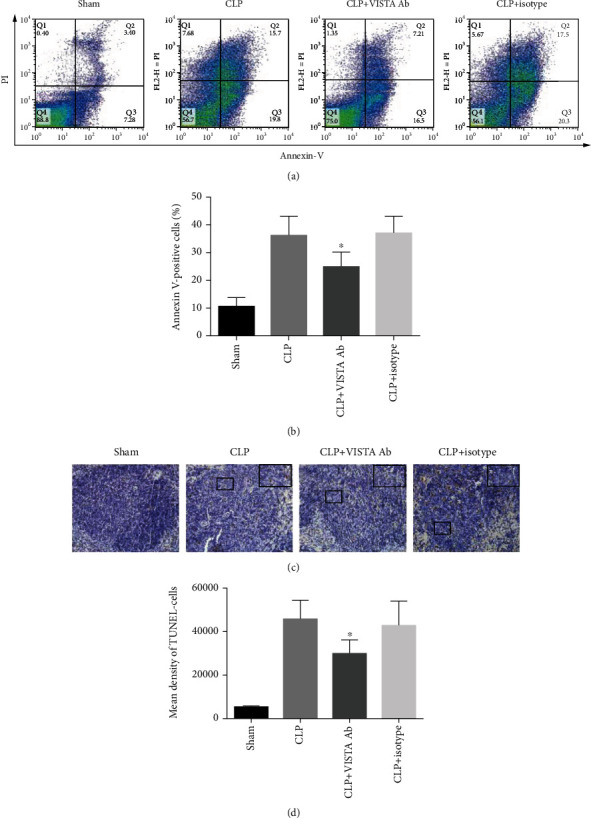
(a) Engagement of VISTA receptor mitigated sepsis-induced apoptosis both in the thymus and spleen. Representative images of CD3^+^ T cell apoptosis in the thymus detected by flow cytometry. (b) Summary data of thymic T cell apoptosis indicated by annexin V-positive cells. Both early apoptotic cells (annexin V-positive/PI-negative) and late apoptotic cells (annexin V-positive/PI-positive) were considered as apoptotic events. Treatment with MH5A decreased the number of annexin V-positive cells in the thymus. (c) Representative images of apoptosis in the spleen detected by TUNEL staining. Sections were examined at a magnification of 400x. (d) Summary data of splenocyte apoptosis indicated by mean density of TUNEL-positive cells. VISTA engagement significantly inhibited sepsis-associated apoptosis in the spleen. All presented data are a composite of three independent experiments (*n* = 6 − 8 in each group). ^∗^*P* < 0.05.

Apoptosis was determined in the spleen and thymus at 24 h post-CLP or sham surgery. In the spleen, TUNEL-positive cells were sporadically found in the sham-operated mice, and the numbers significantly increased after the induction of septic peritonitis. Similarly, the number of annexin V-positive T cells was extremely low in the thymus from the sham group, and sepsis resulted in a marked increase in the number of apoptotic events. Importantly, treatment with MH5A decreased the number of TUNEL-positive cells in the spleen as well as annexin V-positive cells in the thymus (Figure 4).

### 3.6. Anti-VISTA mAb Reversed the Apoptosis of CD3^+^ T Cells In Vitro

**Figure 5:**
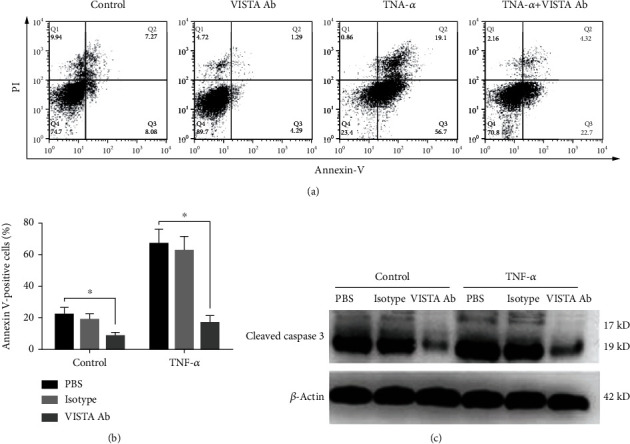
Anti-VISTA mAb reversed TNF-*α*-induced CD3^+^ T cell apoptosis *in vitro*. (a) Representative images of CD3^+^ T cell apoptosis detected by flow cytometry. Spleen T cells were enriched by using CD3-coating immunomagnetic beads and then cultured with or without the presence of MH5A antibody or isotype control. Apoptosis was induced by TNF-*α* (1 *μ*g/mL) stimulation *in vitro*. (b) Summary data of T cell apoptosis indicated by percentage of annexin V-positive cells. MH5A reversed T cell apoptosis cultured from CLP mice with or without the stimulation. ^∗^*P* < 0.05. (c) Expression of cleaved caspase-3 was determined by Western blotting assay. MH5A inhibited cleaved caspase-3 expression cultured from CLP mice with or without TNF-*α* stimulation.

Spleen CD3^+^ T cells were isolated and cultured from CLP or sham-operated mice, and then challenged with or without TNF-*α*. As shown in Figure 5, TNF-*α* induced substantial apoptosis of T cells, as evidenced by increased proportion of annexin V-positive cells and upregulation of cleaved caspase-3. Pretreatment with MH5A antibody substantially reversed apoptosis with or without TNF-*α* stimulation in T cells cultured from the CLP mice. MH5A seemed to have no significant effect on the apoptosis of T cells cultured from sham-operated mice (see Supplement Figure 2).

### 3.7. Anti-VISTA mAb Dampened Cytokine Expression of Macrophage In Vitro

**Figure 6:**
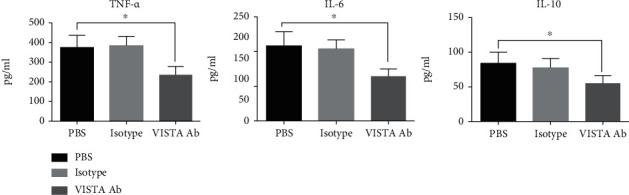
MH5A antibody suppressed LPS-induced cytokine expression on macrophage *in vitro*. Pretreatment with MH5A antibody inhibited the expression of TNF-*α*, IL-6, and IL-10 in peritoneal macrophage stimulated by LPS. All presented data are a composite of three independent experiments (*n* = 8 in each group). ^∗^*P* < 0.05.

LPS induced significantly high expression of TNF-*α*, IL-6, and IL-10 in peritoneal macrophage. Importantly, pretreatment with MH5A antibody suppressed these cytokine expressions. The level of TNF-*α*, IL-6, and IL-10 was significantly lower in the MH5A-treated group, as compared to the control group (Figure 6).

## 4. Discussion

Despite significant progress being made in the understanding of the pathogenesis of sepsis, the underlying mechanisms responsible for the host immune response remain to be elucidated. Numerous studies have revealed the profound effects of sepsis on circulating and tissue T cells. Lymphocyte counts, phenotype, and function were significantly altered in septic patients, which was associated with deleterious outcomes [3]. Recent studies highlighted the close link between the negative immune checkpoints and acquisition of T cell function [13]. Indeed, blockade of PD-1/PD-L1 or CTLA-4 augmented the T cell functions in chronic virus infections, cancer, and clinically relevant models of sepsis [14–16]. In the present study, we found that a high-affinity anti-VISTA Ab improved the survival in a mouse model of septic peritonitis, implying a novel and nonredundant role of this inhibitory molecule in the pathogenesis of sepsis. The therapeutic effects seem to be associated with the inhibition of lymphocyte apoptosis, suppression of inflammatory response, and enhanced bacterial clearance.

Sepsis-induced immune cell apoptosis has been confirmed in several postmortem studies. The apoptosis of immune cells involves CD4^+^ and CD8^+^ T cells, B cells, and dendritic cells in various organs [3, 17, 18]. A very important effect of the MH5A Ab was its reversal of sepsis-induced apoptosis of T cells in the thymus and spleen. Theoretically, treatment with MH5A could preserve the T cell immunity and partially inhibit the apoptosis in septic mice by the suppression of proinflammatory response, but the direct effects of VISTA signaling on T cells might also play an important role. Wang et al. found that VISTA-Ig had minimal direct effects on the apoptosis of cultured T cells without stimulation [8]. In contrast, our study demonstrated that anti-VISTA antibody mitigated the apoptosis of T cells cultured from septic mice to some extent. Further, the antiapoptotic effects were not seen in cultured T cells from sham-operated mice, suggesting that VISTA-mediated apoptosis might require the activation of some other signaling pathways. Although several studies have convincingly demonstrated that engagement of VISTA receptor with VISTA Ig or MH5A Ab can directly suppress T cell function [19–22], intracellular mechanisms underlying VISTA immunomodulation require further elucidation.

It is important to note that the activation of the VISTA pathway substantially inhibits the massive inflammatory response during sepsis. Previous studies have demonstrated that engagement of VISTA receptor could suppress the cytokine expression by T cells [8, 23]. VISTA-Ig downregulated the production of Th1 cytokines, IL-2, and IFN-*γ* from bulk purified CD4^+^ T cell culture [8]. Hence, the decreased cytokine expression in mice treated with MH5A would be partially attributed to the inhibition of T cell response. It needs to be emphasized that the cytokine expression during sepsis was primarily dependent on myeloid cells, and VISTA is highly expressed by these myeloid subsets. A previous study demonstrated that the overexpression of VISTA on monocytes leads to spontaneous secretion of IL-8, IL-1*β*, IL-6, IL-10, and TNF-*α*, suggesting apparent opposing functions of VISTA on T cells and monocytes [24]. On contrast, Han et al. [11] found that VISTA on both T cells and myeloid cells including neutrophils and pDCs could transmit inhibitory signals, resulting in reduced activation and function, establishing VISTA as an inhibitory molecule on myeloid cells. In this study, we found that engagement of VISTA receptor by MH5A antibody significantly inhibited the LPS-induced cytokine expression by macrophage. The molecular structure of VISTA on different immune cells and the crosstalk of receptors/ligands requires further investigation. Another possible explanation for the lessened inflammatory response is that T lymphocyte restoration might facilitate the bacterial clearance and decrease the pathogen loading in vital organs, which in turn reduces the cytokine production in sepsis.

The B7 family represents a class of structurally related coinhibitory molecules, which deliver negative signals to dampen the T cell response. The immune checkpoints such as CTLA-4, PD-1/PD-L1, TIM-3, and LAG-3 are all found to be upregulated on T cells in murine sepsis models, and these coinhibitory molecules have emerged as fundamental targets for reversing sepsis-induced immunosuppression [7]. In particular, the biology of VISTA seems to be distinct from other coinhibitory molecules in the regulation of T cell activation. First, VISTA was abundantly expressed on the resting T cells and macrophage in sham-operated animals, and septic insult did not significantly enhance its expression. Studies from Chen's lab have repeatedly demonstrated that VISTA engagement could suppress the activation of resting T cells through a putative VISTA counter-receptor expression [11, 23]. Given the persistent high expression of VISTA on the immune subsets, it would be plausible to speculate that VISTA engagement could preserve the host immunity since the very early phase of sepsis. Second, VISTA was expressed by both T cell and myeloid cells, and it may act as both a ligand and receptor in regulating the immune response, and these functions are not mutually exclusive. A previous study showed that the optimal T cell activation occurred in the absence of VISTA on both T cell and APCs [23]. Therefore, the anti-VISTA antibody might function to prevent the engagement of unknown counter-receptor or directly by targeting VISTA expressed on T cells and other myeloid cells.

The present study has several limitations. First, in consideration of high expression of VISTA on resting T cells and macrophage, we determined the therapeutic effects of the high-affinity anti-VISTA antibody at one single dose during the early phase of sepsis. Hence, the potential effects in a later phase or the prophylactic treatment for sepsis remain to be elucidated. Second, in this study, we mainly focused on the partial effects of VISTA on the functioning of T lymphocytes and macrophages, while VISTA is expressed on a wider range of hematopoietic cells. The particular role of VISTA in the modulation of other immune cells and T cell-APC interactions during sepsis is warranted in future studies. Finally, the function of anti-VISTA antibody (clone MH5A) has been evaluated in several previous studies which demonstrate MH5A as an agonistic antibody, but the full biological function and downstream signaling of the antibody still requires further elucidation. The full characterization of immune subsets expressing VISTA in sepsis remains to be performed.

## 5. Conclusion

In conclusion, this study identified VISTA as a novel immune checkpoint in the pathogenesis of sepsis. Anti-VISTA antibody protected mice against sepsis by reversing lymphocyte apoptosis and suppressing macrophage activation, suggesting its nonredundant role in controlling immune response during sepsis. VISTA was abundantly expressed at the early stage of sepsis, differing from other B7 family ligands, such as PD-1 and CTLA-4. Thus, targeting VISTA may provide an attractive and novel opportunity in the development of the immunotherapy against sepsis.

## Data Availability

All data in the current study are available from the corresponding authors on reasonable request.

## Supplementary Materials

Supplementary 1 Supplement Figure 1: VISTA expression on T lymphocytes and macrophage during sepsis. (A) Representative image of VISTA expression on CD4^+^ T cells, CD8^+^ T cells, and macrophage at 24 h and 72 h postsurgery. Sham-operated mice were determined at 24 hours postsurgery. The data are representative of 6-8 mice per group from three independent experiments. MFI: mean fluorescence intensity. ^∗^*P* < 0.05. (B) Summary data of VISTA expression on T cells and macrophage at 24 h and 72 h postsurgery. The data are representative of 6-8 mice per group from three independent experiments. MFI: mean fluorescence intensity. ^∗^*P* < 0.05.

Supplementary 2 Supplement Figure 2: effects of anti-VISTA Ab on the T cell apoptosis in the sham group. Cleaved caspase-3 expression in T cells indicated by Western blotting analysis. Anti-VISTA Ab did not affect the cleaved caspase-3 expression with or without TNF-*α* simulation. (A) Summary data of T cell apoptosis indicated by percentage of annexin V-positive cells. In the sham group, VISTA receptor activation has no significant effect on T cell apoptosis. All presented data are a composite of three independent experiments (*n* = 8 in each group). ^∗^*P* < 0.05.

## References

[1] Rhodes A., Evans L. E., Alhazzani W. (2017). Surviving Sepsis Campaign: International Guidelines for Management of Sepsis and Septic Shock: 2016. *Critical Care Medicine*.

[2] Xie J., Wang H., Kang Y. (2020). The epidemiology of sepsis in Chinese ICUs: a national cross-sectional survey. *Critical Care Medicine*.

[3] Boomer J. S., To K, Chang K. C. (2011). Immunosuppression in patients who die of sepsis and multiple organ failure. *JAMA*.

[4] Venet F., Monneret G. (2018). Advances in the understanding and treatment of sepsis-induced immunosuppression. *Nature Reviews Nephrology*.

[5] Fallon E. A., Biron-Girard B. M., Chung C. S. (2018). A novel role for coinhibitory receptors/checkpoint proteins in the immunopathology of sepsis. *Journal of Leukocyte Biology*.

[6] Baksh K., Weber J. (2015). Immune checkpoint protein inhibition for cancer: preclinical justification for CTLA-4 and PD-1 blockade and new combinations. *Seminars in Oncology*.

[7] Wakeley M. E., Gray C. C., Monaghan S. F., Heffernan D. S., Ayala A. (2020). Check point inhibitors and their role in immunosuppression in sepsis. *Critical Care Clinics*.

[8] Wang L., Rubinstein R., Lines J. L. (2011). VISTA, a novel mouse Ig superfamily ligand that negatively regulates T cell responses. *The Journal of Experimental Medicine*.

[9] Le Mercier I., Chen W., Lines J. L. (2014). VISTA regulates the development of protective antitumor immunity. *Cancer Research*.

[10] Wang L., Le Mercier I., Putra J. (2014). Disruption of the immune-checkpoint VISTA gene imparts a proinflammatory phenotype with predisposition to the development of autoimmunity. *Proceedings of the National Academy of Sciences of the United States of America*.

[11] Han X., Vesely M. D., Yang W. (2019). PD-1H (VISTA)-mediated suppression of autoimmunity in systemic and cutaneous lupus erythematosus. *Science Translational Medicine*.

[12] Zhang Y., Zhou Y., Lou J. (2010). PD-L1 blockade improves survival in experimental sepsis by inhibiting lymphocyte apoptosis and reversing monocyte dysfunction. *Critical Care*.

[13] Chen L. (2004). Co-inhibitory molecules of the B7-CD28 family in the control of T-cell immunity. *Nature Reviews Immunology*.

[14] Velu V., Shetty R. D., Larsson M., Shankar E. M. (2015). Role of PD-1 co-inhibitory pathway in HIV infection and potential therapeutic options. *Retrovirology*.

[15] O’Neill R. E., Cao X. (2019). Co-stimulatory and co-inhibitory pathways in cancer immunotherapy. *Advances in Cancer Research*.

[16] Hutchins N. A., Unsinger J., Hotchkiss R. S., Ayala A. (2014). The new normal: immunomodulatory agents against sepsis immune suppression. *Trends in Molecular Medicine*.

[17] Garofalo A. M., Lorente-Ros M., Goncalvez G. (2019). Histopathological changes of organ dysfunction in sepsis. *Intensive Care Medicine Experimental*.

[18] Hotchkiss R. S., Schmieg R. E., Swanson P. E. (2000). Rapid onset of intestinal epithelial and lymphocyte apoptotic cell death in patients with trauma and shock. *Critical Care Medicine*.

[19] Lines J. L., Pantazi E., Mak J. (2014). VISTA is an immune checkpoint molecule for human T cells. *Cancer Research*.

[20] Liu J., Yuan Y., Chen W. (2015). Immune-checkpoint proteins VISTA and PD-1 nonredundantly regulate murine T-cell responses. *Proceedings of the National Academy of Sciences of the United States of America*.

[21] Deng J., Le Mercier I., Kuta A., Noelle R. J. (2016). A new VISTA on combination therapy for negative checkpoint regulator blockade. *Journal for Immunotherapy of Cancer*.

[22] Ohno T., Kondo Y., Zhang C., Kang S., Azuma M. (2017). Immune Checkpoint Molecule, VISTA Regulates T-Cell-Mediated Skin Inflammatory Responses. *Diabetes Care*.

[23] Flies D. B., Han X., Higuchi T. (2014). Coinhibitory receptor PD-1H preferentially suppresses CD4^+^ T cell-mediated immunity. *The Journal of Clinical Investigation*.

[24] Bharaj P., Chahar H. S., Alozie O. K. (2014). Characterization of programmed death-1 homologue-1 (PD-1H) expression and function in normal and HIV infected individuals. *PLoS One*.

