# Supplementary figures and images for: High-Affinity Anti-VISTA Antibody Protects against Sepsis by Inhibition of T Lymphocyte Apoptosis and Suppression of the Inflammatory Response
Source: Mediators Inflamm. 2021 Jul 28;2021:6650329. doi: 10.1155/2021/6650329 (PMC8339895; doi:10.1155/2021/6650329)

**A**

VISTA expression

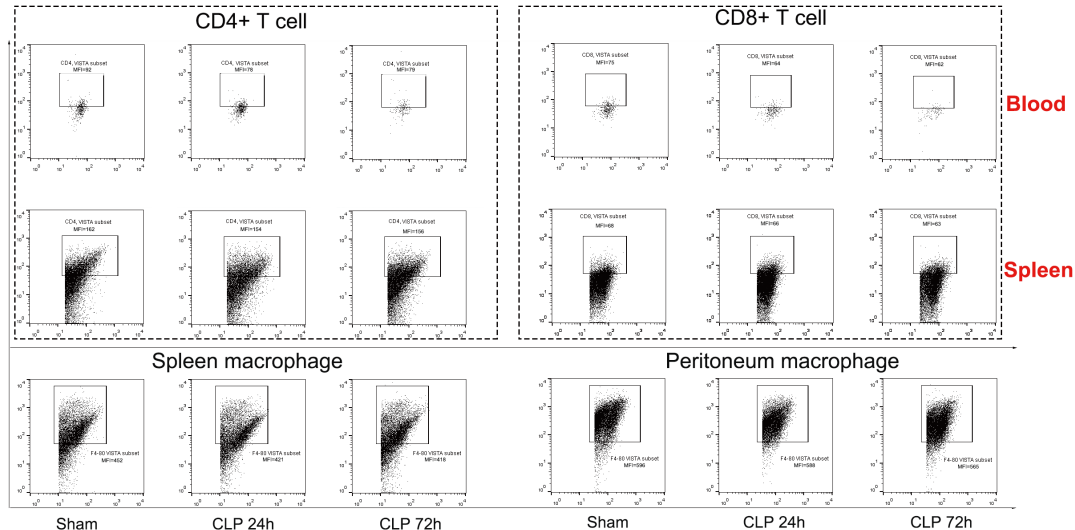**B**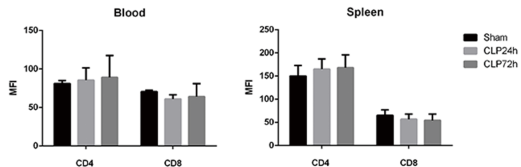**C**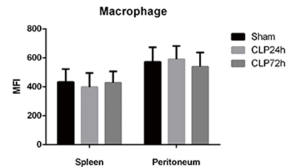

Supplement: Supplementary 1 — Supplement Figure 1: VISTA expression on T lymphocytes and macrophage during sepsis. (A) Representative image of VISTA expression on CD4+ T cells, CD8+ T cells, and macrophage at 24 h and 72 h postsurgery. Sham-operated mice were determined at 24 hours postsurgery. The data are representative of 6-8 mice per group from three independent experiments. MFI: mean fluorescence intensity. ∗P < 0.05. (B) Summary data of VISTA expression on T cells and macrophage at 24 h and 72 h postsurgery. The data are representative of 6-8 mice per group from three independent experiments. MFI: mean fluorescence intensity. ∗P < 0.05. [file 6650329.f1.pdf]

**A**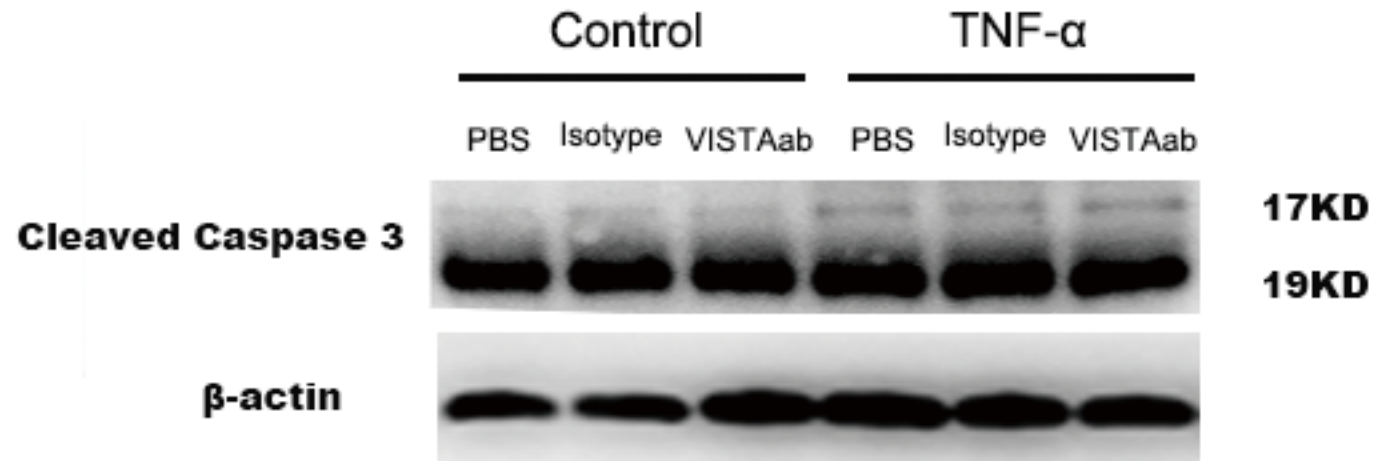**B**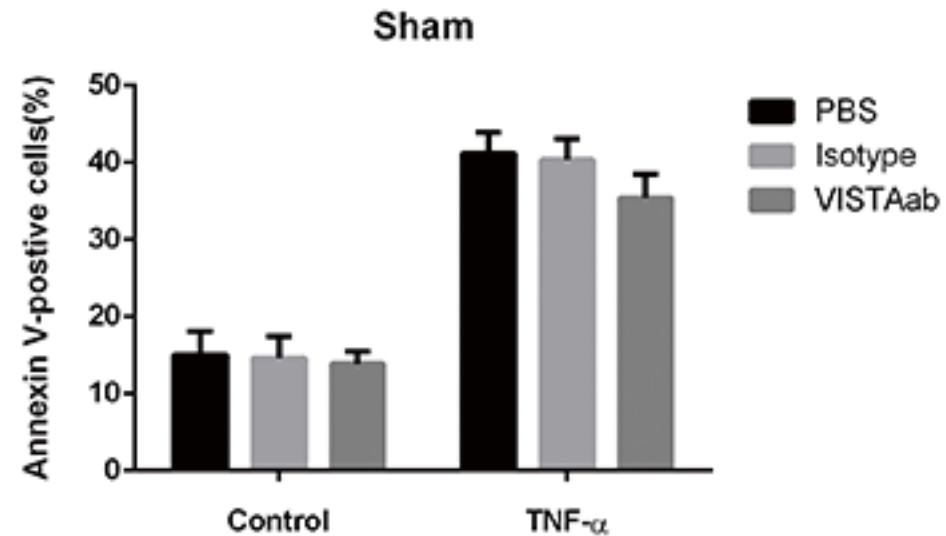

Supplement: Supplementary 2 — Supplement Figure 2: effects of anti-VISTA Ab on the T cell apoptosis in the sham group. Cleaved caspase-3 expression in T cells indicated by Western blotting analysis. Anti-VISTA Ab did not affect the cleaved caspase-3 expression with or without TNF-α simulation. (A) Summary data of T cell apoptosis indicated by percentage of annexin V-positive cells. In the sham group, VISTA receptor activation has no significant effect on T cell apoptosis. All presented data are a composite of three independent experiments (n = 8 in each group). ∗P < 0.05. [file 6650329.f2.pdf]
